# Mathematical Modeling of Systemic Sclerosis and Its Treatment

**DOI:** 10.1007/s11538-026-01700-9

**Published:** 2026-07-28

**Authors:** Teddy Lazebnik, Avner Friedman

**Affiliations:** 1 Department of Information Systems, University of Haifa, Haifa, Israel; 2 Department of Computing, Jonkonping University, Jonkoping, Sweden; 3 Department of Mathematics, The Ohio State University, Columbus, OH USA

**Keywords:** Dermal fibrosis, Reaction–diffusion model, Treatment optimization

## Abstract

Systemic sclerosis (SSc) is an autoimmune disease marked by excessive extracellular matrix (ECM) deposited by myofibroblasts. The disease carries the risk of pathologically progressing to internal organs, particularly the lung. Since abnormally large densities of myofibroblasts are associated with SSc, clinical studies consider trials that reduce the density of myofibroblasts: imatinib treatment (N), which promotes apoptosis in myofibroblasts, and SSc by fresolimumab (S), which inhibits TGF-β, a key growth factor of myofibroblasts. In this paper, we develop a mathematical model of SSc by a system of partial differential equations, and use it to explore a range of treatment strategies with N and S. For example, we considered administering S in fractions three weeks apart. In this case, we determined the smallest amount of S such that ρ(t), the density of the ECM at time t, will continuously and oscillatingly decrease from a disease level $2\rho ^0$, where $\rho ^0$ is the density ρ in health. Since SSc has no cure, ρ(t) cannot decrease below $\rho ^0$. We found that with the smallest amount of S, ρ(t) decreases over a few months to $1.19\rho ^0$ and remains nearly stable thereafter. The results of the paper could be useful in the design of future clinical trials aimed to decrease the excessive extracellular matrix in SSc patients.

## Introduction

Fibrosis is defined as the excessive accumulation of extracellular matrix components, primarily collagen, and fibronectin. It is characterized by overgrowth, hardening and/or scarring of tissue. Scleroderma is a group of fibrotic diseases characterized by thickening and hardening of the skin. Systemic sclerosis (SSc) is a rare autoimmune scleroderma disease (Cleveland Clinic 2023). The disease cannot be cured (Mayo Clinic Staff 2024), and treatments can best decrease the severity of the symptoms (Hopkins Medicine 2019). The disease is not life-threatening, but it can have severe, heterogeneous clinical course. SSc can progress from the skin to internal organs, resulting, for example, in interstitial lung disease (SSc-ILD) (Cottin and Brown 2019); 5 year survival rate for patients with SSc-ILD is 85%–90% (Flavia et al. 2022).

SSc may occur at any age, but most patients develop the disease between the ages of 40 and 50 years (Moinzadeh et al. 2020). The prevalence of SSc worldwide is 200 people per 1 million (van Caam et al. 2018).

The number and shape of fibroblasts do not change in SSc (Zhu et al. 2024; Garrett et al. 2017). By contrast, the number of myofibroblasts is significantly increased. Myofibroblasts are contractile, collagen-secreting cells. They are rare in healthy tissue, but are found in healthy skin, where they originate from fibroblast-to-myofibroblasts transition (Tai et al. 2021). Myofibroblasts population increases in wound healing, where they are needed to close the wound by depositing collagen, a process that results in scarring.

Myofibroblasts have been associated with SSc pathophysiology (van Caam et al. 2018; Tai et al. 2021). Since the etiology of SSc is unknown, experimental and clinical studies have been focusing on targeting myofibroblasts; see (van Caam et al. 2018; Tai et al. 2021) for lists of clinical trials.

TGF-β is constitutively expressed in the skin (Yang et al. 1999), where it is deposited by fibroblasts (Juhl et al. 2020). TGF-β is a central mediator in fibroblast-myofibroblasts conversion (Border and Noble 1994; Vallée and Lecarpentier 2019). TGF-β is secreted by myofibroblasts (Porte et al. 2021), and it is known to significantly increase in SSc (van Caam et al. 2018; Tai et al. 2021). TGF-β is a key growth factor for myofibroblasts formation (van Caam et al. 2018).

Fresolimumab is a rapid inhibitor of TGF-β-regulated gene expression, and has been shown to be effective in the treatment of SSc (Rice et al. 2015).

Myofibroblasts caspase-dependent apoptosis pathway is inhibited by BCL-X binding to pro-apoptosis BIM (van Caam et al. 2018). SSc therapeutic BH3-mimetic drug ABT-263 (navitoclax) displaces BCL-X binding to BIM, allowing BIM to induce apoptosis of myofibroblasts (Lagares et al. 2017).

ABT-263 was shown to significantly reduce dermal thickness in mice model of SSc (Lagares et al. 2017). Imatinib is a drug that targets c-ABL, a protein that activates BCL-X (van Caam et al. 2018); hence, like ABT-263, it induces apoptosis in myofibroblasts. In clinical trials, it was shown that imatinib improved Rodman skin core assessment of skin fibrosis (mRSS) in SSc (Gordon et al. 2014).

In this paper, we develop a mathematical model of SSc and, based on the data from Rice et al. (2015) and Gordon et al. (2014), we use the model to assess the benefit of a variety of treatment protocols by fresolimumab and by imatinib.

## Mathematical Model

The model variables are listed in Table 1 in densities with units of $g/cm^3$. Throughout the model, $d_X$ and $\delta _X$ denote the degradation/death rate and diffusion coefficient of species X, respectively. For production and activation parameters, the first subscript denotes the species being produced or increased, and the second subscript denotes the source or regulator. Thus, $\lambda _{T_\beta F}$ denotes production of $T_\beta$ by fibroblasts, $\lambda _{T_\beta M}$ denotes production of $T_\beta$ by myofibroblasts, $\lambda _{\rho F}$ denotes ECM deposition by fibroblasts, and $\lambda _{\rho M}$ denotes ECM deposition by myofibroblasts. Transition rates are denoted using an arrow; for example, $\lambda _{F\rightarrow M}$ is the basal fibroblast-to-myofibroblast transition rate. The dimensionless parameter $\alpha ^{T_\beta }_{F\rightarrow M}$ denotes the enhancement.

**Table 1:** A list of the model variables

Variable	Definition
F	Fibroblasts
M	Myofibroblasts
$T_\beta$	TGF-β
ρ	ECM density
S	Fresolimumab
N	Imatinib

**Fig. 1:**
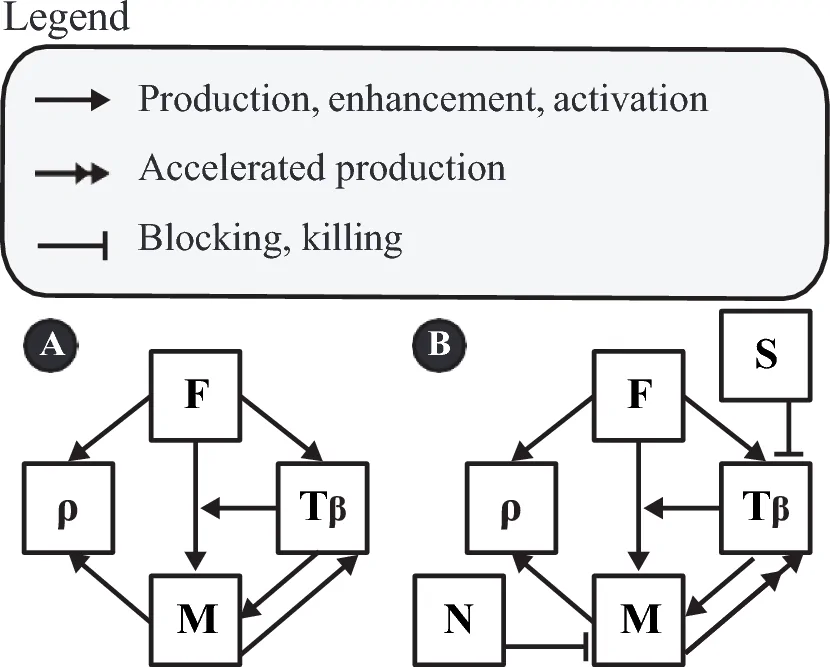
Network of model’s variables in health (A) and in SSc (B)

The mathematical model is based on Fig. 1, and is represented by a system of PDEs. We consider two versions of the model: (A) in health and (B) under the SSc treatment.

We note that fibroblasts secrete collagen, but myofibroblasts secrete collagen more effectively, and they also secrete fibronectin (Baum and Duffy 2011). Since myofibroblast and TGF-β are mutually positively correlated and both are highly present in SSc, we made the assumption that, in SSc, the production of $T_\beta$ by myofibroblasts is accelerated.

We first write down the equations based on Fig. 1(A) (in health).

### Equations for Model (A)

Equation for F. We assume logistic growth for F at rate $\lambda _F$, death rate $d_F$, and fibroblast-to-myofibroblast transition. The transition flux is taken to be

$$\begin{aligned} J_{F\rightarrow M} = \lambda _{F\rightarrow M}F \left( 1+\alpha ^{T_\beta }_{F\rightarrow M} \frac{T_\beta }{K_{T_\beta }+T_\beta } \right) , \end{aligned}$$

where $\lambda _{F\rightarrow M}$ is the basal fibroblast-to-myofibroblast transition rate and $\alpha ^{T_\beta }_{F\rightarrow M}$ is a dimensionless factor describing the enhancement of this transition by $T_\beta$. Hence,

1 $$\begin{aligned} \frac{\partial F}{\partial t} - \delta _F \nabla ^2 F = \lambda _F F\left( 1 - \frac{F}{F_0}\right) - J_{F\rightarrow M} - d_F F, \end{aligned}$$

where $\delta _F$ is the diffusion coefficient of F, and $F_0$ is the carrying capacity of F.

Equation for M. We write the equation for myofibroblasts in the following form:

2 $$\begin{aligned} \frac{\partial M}{\partial t} - \delta _M \nabla ^2 M = J_{F\rightarrow M} + \lambda _{M T_\beta } M \frac{T_\beta }{K_{T_\beta } + T_\beta } - d_M M, \end{aligned}$$

where $\lambda _{M T_\beta }$ is the growth rate of M induced directly by $T_\beta$. This notation follows the convention that the first subscript denotes the species whose equation is affected and the second subscript denotes the regulator. Thus, $\lambda _{M T_\beta }$ refers to $T_\beta$-induced growth of myofibroblasts, whereas $\lambda _{T_\beta M}$, used below in Eq. (3), refers to production of $T_\beta$ by myofibroblasts. The first term on the right-hand side of Eq. (2) is not an independent source of myofibroblasts. Rather, it is the same transition flux $J_{F\rightarrow M}$ that appears with negative sign in Eq. (1) and with positive sign in Eq. (2). The part of $J_{F\rightarrow M}$ proportional to $\alpha ^{T_\beta }_{F\rightarrow M}$ therefore represents the $T_\beta$-induced increase in the fibroblast-to-myofibroblast transition rate, whereas $\lambda _{M T_\beta }M T_\beta /(K_{T_\beta }+T_\beta )$ represents proliferation/expansion of the existing myofibroblast population in response to $T_\beta$.

Equation for $T_\beta$. $T_\beta$ is produced by both fibroblasts and myofibroblasts, so that:

3 $$\begin{aligned} \frac{\partial T_\beta }{\partial t} - \delta _{T_\beta } \nabla ^2 T_\beta = \lambda _{T_\beta F} F + \lambda _{T_\beta M} M - d_{T_\beta } T_\beta , \end{aligned}$$

where $\lambda _{T_\beta F}$ and $\lambda _{T_\beta M}$ are production rates, $d_{T_\beta }$ is a degradtion rate, and $\delta _{T_\beta }$ is the diffusion coefficient of $T_\beta$.

Equation for ρ. ECM is deposited by fibroblasts and myofibroblasts at rates $\lambda _{\rho F}$ and $\lambda _{\rho M}$, respectively. ECM turnover is represented by the effective first-order term $d_\rho \rho$. This term should be interpreted as a lumped degradation/remodeling rate, which includes protease-mediated degradation of ECM components, rather than as spontaneous degradation. In particular, fibroblasts and other stromal/immune cells can contribute to collagen and ECM degradation through matrix metalloproteinases (MMPs), and fibrosis has been associated not only with excessive ECM production but also with impaired ECM degradation and altered MMP/TIMP balance (Cox et al. 2006; Zhao et al. 2022, 2023; Mayorca-Guiliani et al. 2025). Hence,

4 $$\begin{aligned} \frac{\partial \rho }{\partial t} = \lambda _{\rho F} F + \lambda _{\rho M} M - d_{\rho } \rho . \end{aligned}$$

Note that the connected tissue is not diffusing, hence we do not include diffusion for ρ. A more detailed formulation could include an explicit fibroblast-mediated ECM degradation term, for example $-\lambda _{\rho F}^{\textrm{deg}}F\rho$. However, near the healthy steady state, $F \approx F^0$, so this term is mathematically absorbed into the effective degradation coefficient:

$$ -d_\rho \rho -\lambda _{\rho F}^{\textrm{deg}}F\rho \approx -\left( d_\rho +\lambda _{\rho F}^{\textrm{deg}}F^0\right) \rho \equiv -d_\rho ^{\textrm{eff}}\rho . $$

Thus, in the absence of independent measurements of MMP/TIMP activity or collagen degradation products, $d_\rho$ and $\lambda _{\rho F}^{\textrm{deg}}$ cannot be reliably estimated separately from the clinical data used in this study. We therefore retain the compact effective degradation term $d_\rho \rho$.

### Equations for Model (B)

Based on Fig. 1(B) (for SSc with treatment), Eqs. (1) and (4) remain the same as for model (A), but Eqs. (2) and (3) change, as follows:

5 $$\begin{aligned} \frac{\partial M}{\partial t} - \delta _M \nabla ^2 M = J_{F\rightarrow M} + \lambda _{M T_\beta } M \frac{T_\beta }{K_{T_\beta } + T_\beta } - d_M M - d_{MN} MN, \end{aligned}$$

and

6 $$\begin{aligned} \frac{\partial T_\beta }{\partial t} - \delta _{T_\beta } \nabla ^2 T_\beta = \lambda _{T_\beta F} F + \lambda ^*_{T_\beta M} M - d_{T_\beta } T_\beta - d_{T_\beta S} T_\beta S, \end{aligned}$$

where $d_{MN}$ is the killing rate of M by drug N, $d_{T_\beta S}$ is the effective loss rate of $T_\beta$ due to interaction with drug S, and $\lambda ^*_{T_\beta M}$ is a large parameter representing aberrant production of $T_\beta$ by myofibroblasts in SSc.

We write the equation for S as follows:

7 $$\begin{aligned} \frac{\partial S}{\partial t} - \delta _S \nabla ^2 S = c_S h(t) - d_{S T_\beta }S T_\beta - d_S S, \end{aligned}$$

where $\delta _S$ is the diffusion coefficient, $d_S$ is the washout rate of S, $d_{S T_\beta }$ is the effective loss rate of free S due to interaction with $T_\beta$, and $c_S$ is the dose amount of the drug. The two parameters $d_{T_\beta S}$ and $d_{S T_\beta }$ describe the two sides of the same effective interaction between fresolimumab and $T_\beta$. The term $d_{T_\beta S}T_\beta S$ appears in the $T_\beta$ equation because it represents loss of free $T_\beta$, whereas the term $d_{S T_\beta }S T_\beta$ appears in the S equation because it represents loss of free fresolimumab. Since independent measurements of these two effective rates are not available, we take $d_{T_\beta S}=d_{S T_\beta }$ in the simulations. If the drug is administrated at days $t_0 = 0, t_1, t_2,..., t_k$, then:

$$\begin{aligned} h(t) = {\left\{ \begin{array}{ll} e^{-\nu t} & \text {for } 0 \le t< t_1 \\ e^{-\nu t} + e^{-\nu (t-t_1)} & \text {for } t_1 \le t < t_2 \\ . \\ . \\ . \\ e^{-\nu t} + e^{-\nu (t-t_1)} + \dots + e^{-\nu (t-t_k)} & \text {for } t > t_k; \\ \end{array}\right. } \end{aligned}$$

the exponential parameter ν is given by $ln(2)/t_{1/2}$ where $t_{1/2}$ is the half-life of the drug.

When N=S=0, the equation for ρ remains the same as in Eq. (4). In SSc, the effective degradation component of ECM turnover may be reduced because fibrosis is associated with impaired collagen/ECM degradation and altered MMP/TIMP regulation (Jinnin 2022; Zhao et al. 2022; Mayorca-Guiliani et al. 2025). In the present model, this process is not represented as a separate biological variable. Instead, $d_\rho$ is treated as an effective turnover parameter and the SSc phenotype is generated through the myofibroblast–TGF-β axis, which is the main treatment target considered here. A model that separately tracks MMPs, TIMPs, and collagen degradation products would require additional data for parameter estimation and is left for future work. But, when N=S=0, the equation for ρ remains the same as in Eq. (4). When treatment is applied, we distinguish between the healthy baseline ECM density, $\rho ^0$, and the pathological excess ECM, $\rho -\rho ^0$. Since SSc has no cure, treatment is assumed to reduce the excess fibrotic ECM but not to eliminate the healthy ECM scaffold. We therefore add a phenomenological singular depletion term acting on the excess ECM. This term is defined on the admissible domain $\rho >\rho ^0$ and represents tissue-level resistance/homeostasis as ρ(t) approaches the healthy ECM level. We accordingly take

8 $$\begin{aligned} \frac{d\rho }{dt} = \lambda _{\rho F} F + \lambda _{\rho M} M - d_\rho \rho - \frac{\epsilon }{\sqrt{\rho - \rho ^0}}, \end{aligned}$$

with drug resistance parameter ϵ. Singular and non-Lipschitz terms are commonly used in mathematical models when the modeled quantity approaches a limiting admissible state. For example, finite-time extinction in diffusion–absorption equations is obtained through strong absorption terms such as $u^q$, 0<q<1, and ODE systems with dissipation terms of negative homogeneity exhibit analogous finite-time extinction behavior (Iagar 2022; Hoang 2025). Singular potentials are also used in phase-field models of tumor growth to encode constraints on biological state variables and keep the solution within an admissible physical range (Colli et al. 2021; Scarpa and Signori 2021). In the present simulations, ρ(t) remains above $\rho ^0$, so the singular term is used only in its intended domain.

### Boundary and Initial Conditions

We consider the model equations in a portion of the skin:

$$ \Omega = \{ (x_1, x_2, x_3); 0 \le x_i \le h, |x_2| \le 2, |x_3| \le 2 \}, $$

where the surface of the skin is in the plane $x_1 = h$; we take an intermediate thickness of the skin (epidermis + dermis) h=0.2 cm (Branchet et al. 1990).

We take the following boundary conditions:

9 $$\begin{aligned} \begin{array}{l} X = 0 \;\; \text {on} \;\; x_1 = h, \\ \\ \partial X/\partial n = 0 \;\; \text {on the remaining parts of the boundary} \;\; X = F, M, T_\beta , \text { and } \\ \\ \partial S/\partial n = 0 \;\; \text {on all the boundary of the domain}, \end{array} \end{aligned}$$

where $\partial /\partial n$ is the normal to the boundary.

We take the following initial conditions at t=0, in model A and in model B with no drug:

10 $$\begin{aligned} F = F^0, \; M = M^0, \; T_\beta = T_\beta ^0, \; \rho = \rho ^0, \end{aligned}$$

where $F^0, M^0, T_\beta ^0$, and $\rho ^0$ are “steady state” values in health, given in Table 2.

**Table 2:** Model parameters: symbols, descriptions, values, and sources

Symbol	Description	Value	Source
$\lambda _F$	Proliferation rate of fibroblasts	$2.06~\textrm{d}^{-1}$	Estimated
$d_F$	Death rate of fibroblasts	$8.3 \cdot 10^{-1} ~\textrm{d}^{-1}$	Seaman et al. (2015)
$\lambda _{F\rightarrow M}$	Basal fibroblast-to-myofibroblast transition rate	$1.0 \cdot 10^{-1}~\textrm{d}^{-1}$	Estimated
$\delta _F$	Diffusion coefficient of fibroblasts	$8.64 \cdot 10^{-7}~\mathrm {cm^2/d}$	Hao et al. (2014)
$F_0$	Carrying capacity of fibroblasts	$15 \cdot 10^{-3}~\mathrm {g/cm^3}$	Estimated
$F^0$	Steady-state concentration of fibroblasts	$7.5 \cdot 10^{-3}~\mathrm {g/cm^3}$	Miller et al. (2003); Padovan-Merhar et al. (2015)
$d_M$	Death rate of myofibroblasts	$1.0 \cdot 10^{0}~\textrm{d}^{-1}$	Estimated
$\delta _M$	Diffusion coefficient of myofibroblasts	$8.64 \cdot 10^{-7}~\mathrm {cm^2/d}$	Hao et al. (2014)
$M^0$	Steady-state concentration of myofibroblasts	$2.5 \cdot 10^{-3}~\mathrm {g/cm^3}$	This work
$\lambda _{M T_\beta }$	$T_\beta$-induced proliferation of myofibroblasts	$8.0 \cdot 10^{-1} ~\textrm{d}^{-1}$	Estimated
$\alpha ^{T_\beta }_{F\rightarrow M}$	Dimensionless enhancement of fibroblast-to-myofibroblast transition by $T_\beta$	$2.0 \cdot 10^{0}$	This work
$d_{MN}$	Killing rate of myofibroblasts by drug *N*	$28.42 \cdot 10^{3} (cm^3/g)/d$	Gordon et al. (2014) fitted
$\delta _{T_\beta }$	Diffusion coefficient of $T_\beta$	$14.8 \cdot 10^{-2}~\mathrm {cm^2/d}$	Young et al. (1980); Liao et al. (2014); Hornbeck et al. (2015)
$d_{T_\beta }$	Degradation rate of $T_\beta$	$4.99\cdot 10^{2}~\textrm{d}^{-1}$	Wakefield et al. (1990)
$T^0_\beta$	Steady-state concentration of $T_\beta$	$3.5 \cdot 10^{-8}~\mathrm {g/cm^3}$	Yang et al. (1999)
$K_{T_\beta }$	Half-saturation constant for $T_\beta$	$3.5 \cdot 10^{-8}~\mathrm {g/cm^3}$	Estimated
$\lambda _{T_\beta F}$	$T_\beta$ production rate by fibroblasts	$1.16 \cdot 10^{-3}~\textrm{d}^{-1}$	Estimated
$\lambda _{T_\beta M}$	$T_\beta$ production rate by myofibroblasts	$5.74 \cdot 10^{-3}~\textrm{d}^{-1}$	Estimated
$\lambda ^*_{T_\beta M}$	Aberrant $T_\beta$ production rate by myofibroblasts in SSc	$1.886 \cdot 10^{1}$ $d^{-1}$	Fitted
$d_{T_\beta S}$	Effective loss of $T_\beta$ due to fresolimumab	$1.6 \cdot 10^3 (cm^3/g)/d$	Estimated
$d_{S T_\beta }$	Effective loss of fresolimumab due to interaction with $T_\beta$	$1.6 \cdot 10^3 (cm^3/g)/d$	Fitted
$d_S$	Washout rate of drug S	$1.0 \cdot 10^{-2}~\textrm{d}^{-1}$	This work
$\delta _S$	Diffusion coefficient of S	$5.0 \cdot 10^{-2}~\textrm{cm}^{2}/d$	Young et al. (1980); Liao et al. (2014), [44]
ν	Exponental decay parameter of S	$3.4 \cdot 10^{-2}/d$	Fitted
$\rho ^0$	Steady-state ECM concentration	$2.1 \cdot 10^{-1} ~\mathrm {g/cm^3}$	National Institute of Standards and Technology (2024); Téllez-Soto et al. (2021); Oikarinen (1994)
$d_\rho$	Degradation rate of ECM	$3.7 \cdot 10^{-1}~\textrm{d}^{-1}$	Xue et al. (2009)
$\lambda _{\rho F}$	ECM deposition rate by fibroblasts	$5.18 \cdot 10^{0}~\textrm{d}^{-1}$	Estimated
$\lambda _{\rho M}$	ECM deposition rate by myofibroblasts	$1,554 \cdot 10^{1}~\textrm{d}^{-1}$	Estimated
ϵ	Phenomenological resistance/homeostatic parameter for excess ECM removal	$1.75 \cdot 10^{3}$	Fitted

## Parameter Estimates

We first estimate the parameters from Model (A) (in health).

### Steady State

We denote by $X^0$ the steady state in health of species X, and assume that in steady state $X/(K_X + X) = 0.5$, where $K_X$ is the (so-called) half-saturation of X; hence $K_X = X^0$.

The number of fibroblast cells in the dermis is 2100–4100 per $mm^3$, with an average of $3000/mm^3$ (Miller et al. 2003). The volume of a fibroblast cell is $2.5 \cdot 10^{-9} cm^3$ (Padovan-Merhar et al. 2015) (Fig. 1B) and its mass is accordingly taken to be $2.5 \cdot 10^{-9}g$. Hence, $F^0 = 7.5 \cdot 10^{-3} g/cm^3$. We assume that in health, $M^0 < F^0$ and take $M^0 = F^0/3 = 2.5 \cdot 10^{-3} g/cm^3$. The skin concentration of $T_\beta$ in health is $30--39\,g/mm^3$ (Yang et al. 1999). We take average of $35\,g/mm^3$, so that $T^0_\beta = 3.5 \cdot 10^{-8} g/cm^3$ and $K_{T_\beta } = 3.5 \cdot 10^{-8} g/cm^3$.

### Death and Degradation Rates

We denote by $t_{1/2}(X)$ the half-life of species X, and use the formula $d_X = ln(2)/t_{1/2}(X)$.

Human fibroblast cell-cycle is between 16 and 28 h, with a mean of 20 h (Seaman et al. 2015). Hence, $t_{1/2}(F) = 20/24 d^{-1}$, and $d_F = ln(2)/(20/24) = 0.83 d^{-1}$. We assume that $d_M > d_F$, and take $d_M = 1.0 d^{-1}$. The half-life of $T_\beta$ is approximately 2 min (Wakefield et al. 1990). Hence, $t_{1/2}(T_\beta ) = 1.39 \cdot 10^{-3} d$, and $d_{T_\beta } = 499 d^-1$.

### Diffusion Coefficients

We take $\delta _F = 8.64 \cdot 10^{-7} cm^2d^{-1}$ from Hao et al. (2014), and $\delta _M = \delta _F$. To estimate the diffusion coefficient of $T_\beta$, we use the formula from Young et al. (1980): $\delta _X = const./m_x^{1/3}$ for any protein X, where $m_x$ is the molecular weight of X; the constant is computed from the data for VEGF (V) in Liao et al. (2014): $\delta _V = 8.54 \cdot 10^{-2} cm^2d^{-1}$ and $m_v = 24kDa$. Since $m_{T_\beta } = 4.76 kDa$ (Hornbeck et al. 2015), we get $\delta _{T_\beta } = 14.8 \cdot 10^{-2} cm^2d^{-1}$.

### Estimate from Equations

We use the steady state of an equation, by taking the right-hand side equal to zero and $X = X^0$ for any species X in the equation.

Equation (2). Taking $\alpha ^{T_\beta }_{F\rightarrow M}=2$, and using $\frac{T_\beta ^0}{K_{T_\beta }+T_\beta ^0}=\frac{1}{2}$, the transition contribution in the healthy steady state is $\lambda _{F\rightarrow M}F^0 \left( 1+\frac{1}{2}\alpha ^{T_\beta }_{F\rightarrow M} \right) = 2\lambda _{F\rightarrow M}F^0$. The steady-state condition for Eq. (2) is therefore $2\lambda _{F\rightarrow M}F^0 + 0.5\lambda _{M T_\beta }M^0 = d_M M^0$. Using $\lambda _{F\rightarrow M}=0.1~d^{-1}$, $d_M=1~d^{-1}$, and $F^0=3\,M^0$, we obtain $0.6M^0+0.5\lambda _{M T_\beta }M^0=M^0$, and hence $\lambda _{M T_\beta }=0.8~d^{-1}.$ In SSc, the production of $T_\beta$ by M is increased compared to the healthy case (see Fig. 1(B)), which means that the aberrant production parameter $\lambda ^*_{T_\beta M}$ is larger than the healthy production parameter $\lambda _{T_\beta M}$. In Medsger and Benedek (2019), skin thickness in SSc was assessed by mRSS. The average thickness depends on the progression of the disease. We accordingly assume that by 168 days the density ρ(t) reaches the level $2\rho ^0$, and by simulation of the model we found that

$$\begin{aligned} \lambda ^*_{T_\beta M}=1.886 \cdot 10^{1}~d^{-1}. \end{aligned}$$

Equation (3). In steady state, $\lambda _{\rho F} F^0 + \lambda _{T_\beta M} M^0 = d_{T_\beta } T_\beta ^0 = 499 \cdot 3.5 \cdot 10^{-8}$. We assume that $\lambda _{T_\beta } F^0 = \lambda _{T_\beta M} M^0$, so that $\lambda _{T_\beta } = 3\lambda _{T_\beta F}$. Recalling that $F^0 = 7.5 \cdot 10^{-3} g/cm^3$, we get $\lambda _{T_\beta F} = 1.16 \cdot 10^{-3} d^{-1}$ and $\lambda _{T_\beta M} = 5.74 \cdot 10^{-3} d^{-1}$.

Equation (4). We take $d_\rho = 0.37 d^{-1}$ from Xue et al. (2009). Skin density is $1.1 g/cm^3$ (National Institute of Standards and Technology 2024), and the total water content is 74% of the dermis (Téllez-Soto et al. 2021). Collagen makes 75% of the dry weight of the skin (Oikarinen 1994). Hence, the density of ECM in healthy skin is $\rho ^0 = 1.1 \cdot 26/100 \cdot 75/100 = 0.21g/cm^3$. In steady state, $\lambda _{\rho F}F^0 + \lambda _{\rho M} M^0 = d_{\rho } \rho ^0 = 7.77 \cdot 10^{-2}$.

According to Baum and Duffy (2011), $\lambda _{\rho M} > \lambda _{\rho F}$, and we take $\lambda _{\rho M} = 3 \lambda _{\rho F}$. Hence, $2 \lambda _{\rho F} F^0 = 7.77 \cdot 10^{-2}$, so that $\lambda _{\rho F} = 5.18 \cdot d^{-1}$ and $\lambda _{\rho M} = 15.54 d^{-1}$.

### Drug-associated Parameters from Model (B)

The molecular weight of S is 144Kd [44].. Hence, by Young et al. (1980); Liao et al. (2014), $\delta _S = 5.0 \cdot 10^{-2} cm^2/d$. We take $\alpha ^{T_\beta }_{F\rightarrow M}=2$ and $d_{MN} = 28.42 \cdot 10^{3} (cm^3/g)/d$ in order to fit model simulation of ρ to the clinical trials in Gordon et al. (2014) described in section 4.2. The molecular weight of fresolimumab is 145kDa, and we accoridng take $\delta _S = 5\cdot 10^{-2} cm^2/d$. The half-life of S is in the range of 14–22 days (Trachtman et al. 2011). Hence, $\nu = ln(2)/t_{1/2}$ is in the range of $3.1 \cdot 10^{-2}< \nu < 4.9 \cdot 10^{-2}$. We assume that $d_S = 1 \cdot 10^{-2}~d^{-1}$ and, because independent measurements of the two sides of the fresolimumab–$T_\beta$ interaction are not available, we take $d_{T_\beta S}=d_{S T_\beta }$. In SSc, ρ(t) is approximately equal to the density of myofibroblasts. We accordingly randomly varied ν, $d_{S T_\beta }$ and the day in week 4 when the second dose of S was injected in Rice et al. (2015) Fig. 3, in order to get the best fit (in 100 days) of ρ(t) with the fold change of cartilage oligomeric protein (COMP), which we take to represent the myofibroblasts density, hence ρ(t). We found that $\nu = 3.4 \cdot 10^{-2}/d, \; d_{S T_\beta } = 1.6 \cdot 10^{3} (cm^3/g)/d$ and the optimal day is 23. We next choose $\epsilon = 1.75 \cdot 10^{-3}$ in Eq. (8) to further optimize the average coefficient of determination (i.e., the fit) between the proposed model simulation and Fig. 3 in Rice et al. (2015), using the Newton–Raphson algorithm (Robert et al. 1976). Table summarizes the model’s paraemters and their values.

## Results

The PDE model takes a second-order and nonlinear form with a free boundary cube geometric configuration. We solve it numerically using the Runge–Kutta method (Verwer and Sommeijer 2004); all parameter values are taken from Table 3.5. In particular, all the numerical analysis in this study was performed using the Python programming language (Langtangen and Logg 2016).

We define the average density of each species X at time t by $X(t) = \frac{1}{3.2}\int _{x \in \Omega } X(t,x)dx$ where X(t,x) is the density of X at (t,x) and $3.2cm^3$ is the volume of Ω.

SSc is an autoimmune disease that has no cure, but treatment can decrease the severity of symptoms and reduce the risk of SSc progressing from the skin to internal organs, particularly to interstitial lung disease. We can use the mathematical model to devise strategies for long-term treatment with N or S to minimize the burden of SSc.

### SSc in the Control Case (No Drugs)

**Fig. 2:**
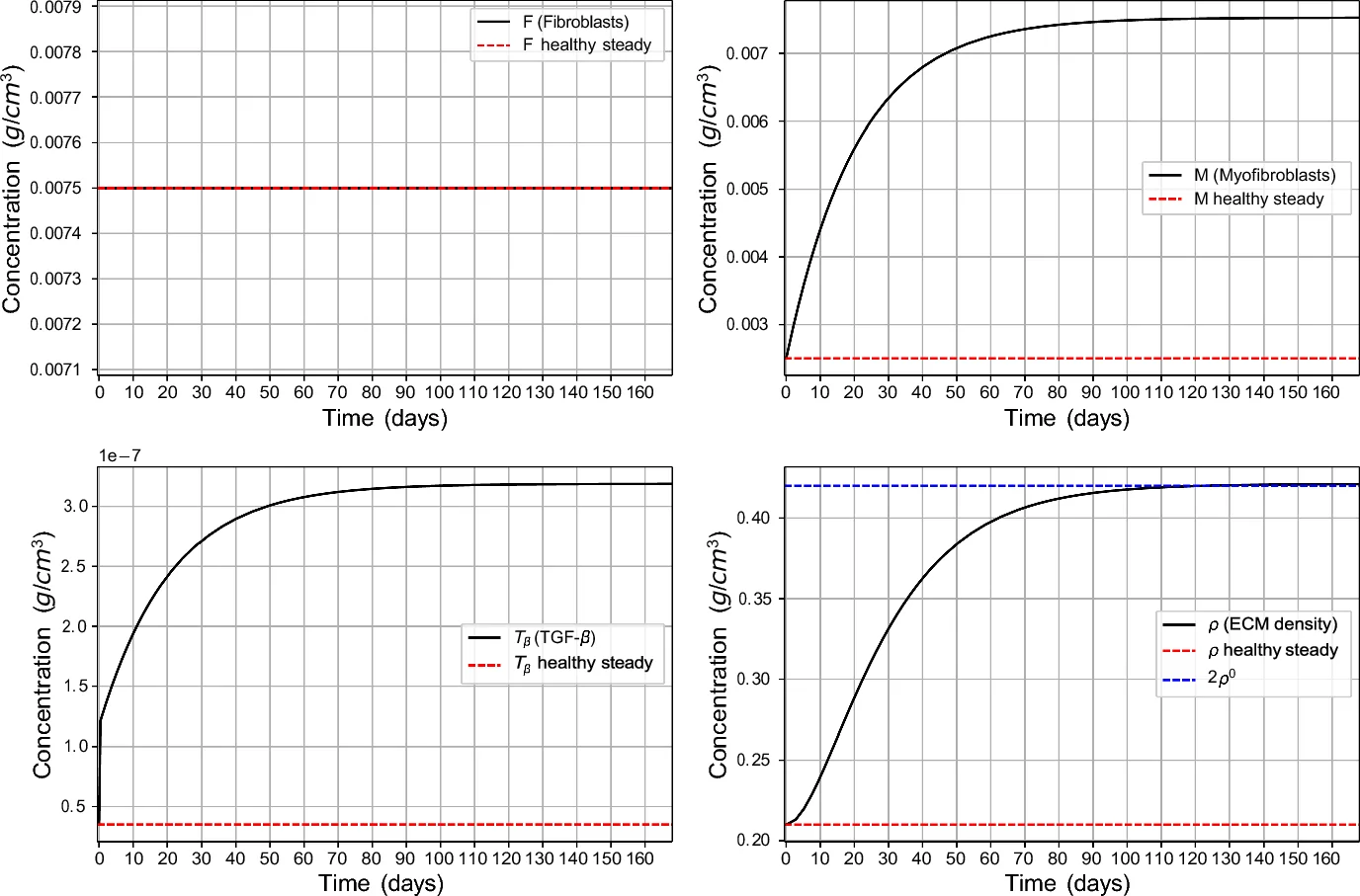
Average densities/concentrations, in $g/cm^3$, of all the model variables, in the case of SSc with no drugs (Color figure online)

In Fig. 2 we simulated the model variables in the control case, i.e., model (B) with N=0,S=0 in Eqs. (5) and (6). We note that the profile of F remains the same as in the healthy case, in agreement with Zhu et al. (2024); Garrett et al. (2017). On the other hand, the densities of $T_\beta$ and myofibroblast are significantly increasing compared to the healthy case, and ρ(t) increases to $2\rho ^0$ as t→168 days (24 weeks).

### SSc Treatment with Imatinib (N)

We consider model (B) with S=0. In clinical trial (Gordon et al. 2014), patients were treated with imatinib from 100 to 400 mg daily by mouth for a period of 12 months. The improvement in mRSS increased linearly in time ( Gordon et al. 2014, Fig. 1), and was 22.4% after 12 months. All patients were initially treated with 400 mg daily, but 87% required at least one drug adjustment because of adverse effects. The median daily dose of the patients was 300 mg. We assume that patients’ average daily dose was 240 mg, and that the human average weight is 80 kg. Taking average tissue density of 1 g per $cm^3$, we arrive at daily drug dose of $N = 3\cdot 10^{-6}\ g/cm^3$.

**Fig. 3:**
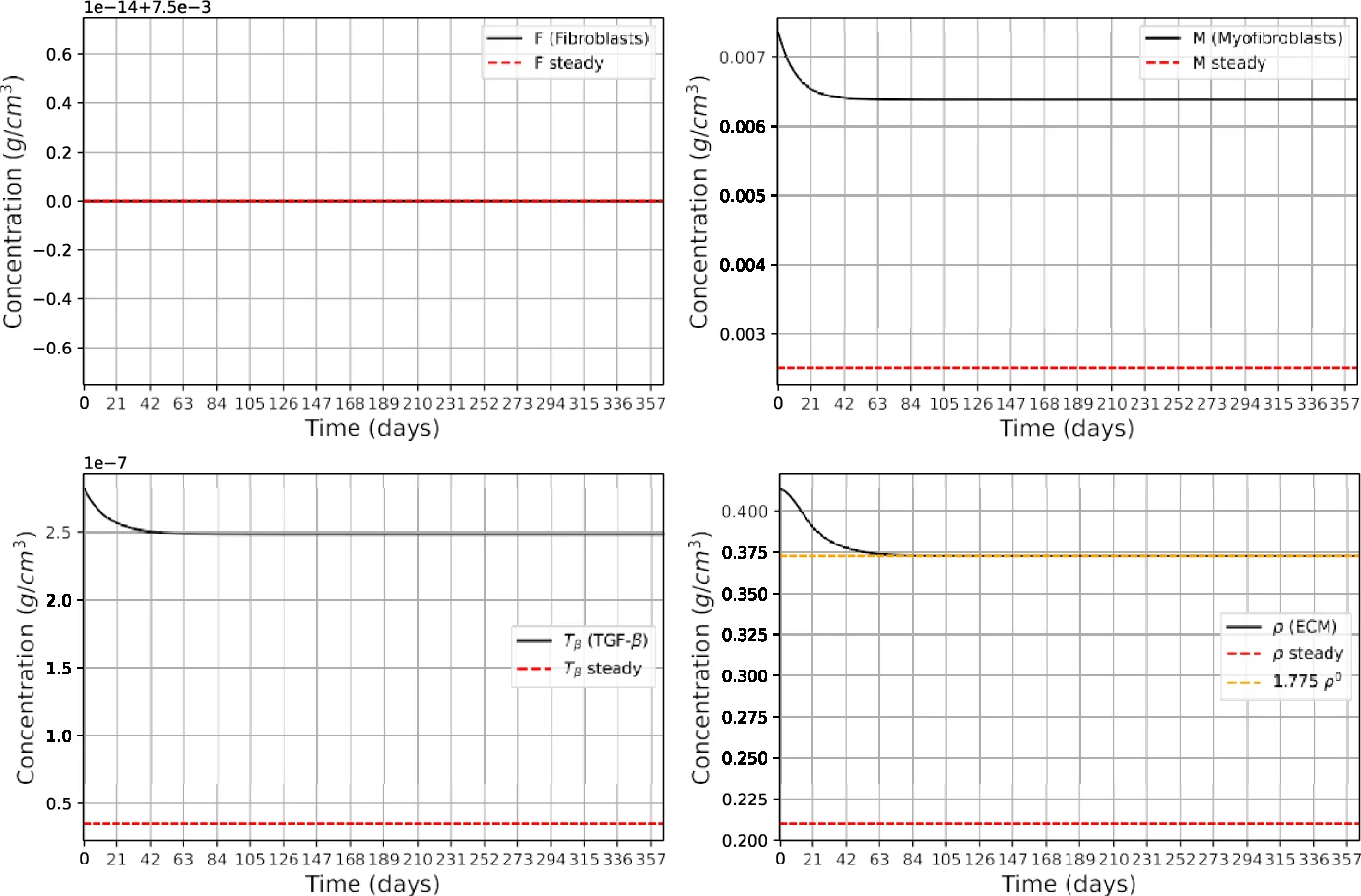
Average densities/concentrations, in $\mathrm {g/cm^3}$, of all the model variables for the case of SSc treatment with drug N (Color figure online)

Fig. 3 shows simulations of the model variables under treatment with N, where we searched for the parameter $d_{MN}$ in the term $d_{MN}MN$ of Eq. (5)). In the clinical trial (Gordon et al. 2014), the improvement in mRSS after 12 months was 22.4%. In the model, we interpret this clinical improvement as a reduction in the pathological excess ECM, $\rho (t)-\rho ^0$, rather than as a 22.4% reduction in the total ECM density ρ(t). Thus, starting from the disease level $\rho (0)=2\rho ^0$, the calibration target is $\rho (365) = \rho ^0+0.776(2\rho ^0-\rho ^0) = 1.776\rho ^0$. Since $\rho ^0=0.21\,\mathrm {g/cm^3}$, this gives $\rho (365) = 1.776\rho ^0 \approx 0.373\,\mathrm {g/cm^3}$. With this calibration, we found that $d_{MN}=28.42\cdot 10^3\,(\mathrm {cm^3/g})/\textrm{d}$.

Fig. 3 also shows that the effect of the drug N, using the assumed average daily dose $N=240\,\textrm{mg}$, is to reduce the pathological excess ECM, $\rho (t)-\rho ^0$, close to the calibrated 22.4% reduction within the first 60 days, after which ρ(t) remains approximately at the same plateau. The percentage reduction in the total ECM density ρ(t) is smaller, because the healthy baseline ECM density $\rho ^0$ is not assumed to be removed by treatment.

**Fig. 4:**
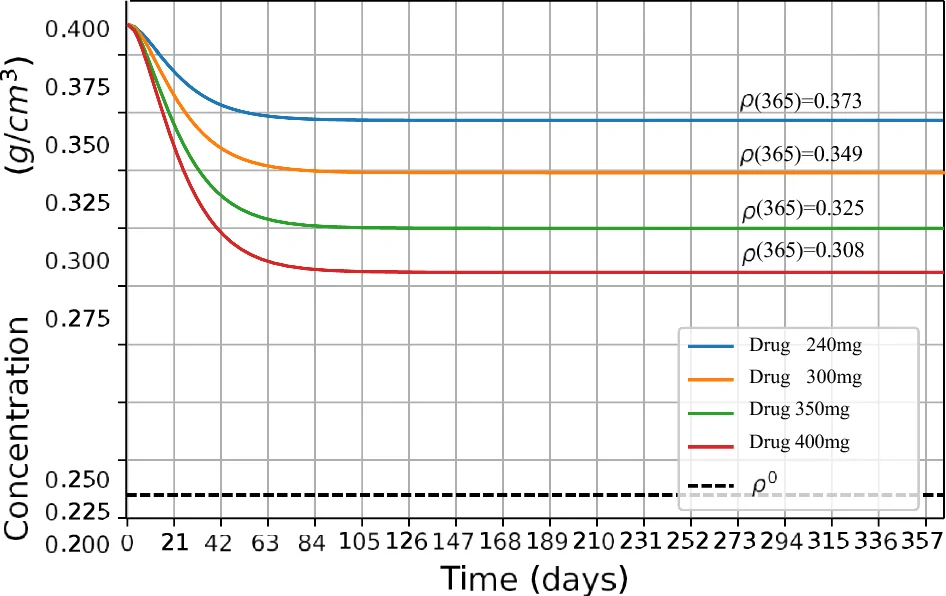
Effect of different doses of the drug N on ρ (Color figure online)

In Gordon et al. (2014), N was administered at dose levels 100–400 mg for different patients, while in Fig. 3, we took the daily dose level to be 240 mg for all patients. This interpretation also explains why the decrease seen in Fig. 2 appears smaller than 22.4% when measured relative to the total ECM density ρ(t): the 22.4% calibration is applied only to the excess fibrotic component $\rho (t)-\rho ^0$. Since 13% of patients in Gordon et al. (2014) were safely treated daily with 400 mg of imatinib, it is interesting to see the effect of the drug when the dose is increased from 240 to 400 mg. In Fig. 4, we took 4 levels of N, namely: 240, 300, 350, and 400 mg, and simulated the profiles of ρ(t) for 2 years. We see that the effect of the drug, in each case, is to quickly reduce ρ(t) to a certain level, and to keep it at this level thereafter.

### SSc Treatment with Fresolimumab (S)

Cartilage oligomeric matrix protein (COMP) is a structural component of cartilage, and studies has described COMP as a pathological factor that promotes collagen deposition in fibrotic skin disorders such as scleroderma [44]. We accordingly consider ρ(t) in SSc to be proportional to COMP gene expression. We take model B with N=0 and follow the clinical trials in Rice et al. (2015). We consider two treatments of SSc patients:

**Treatment 1.** Drug S is given at days 1 and 25 at dose $c_S = 1\,mg/kg$.

**Treatment 2.** Drug S is given just at day 1 at dose $c_S = 5\,mg/kg$.

**Fig. 5:**
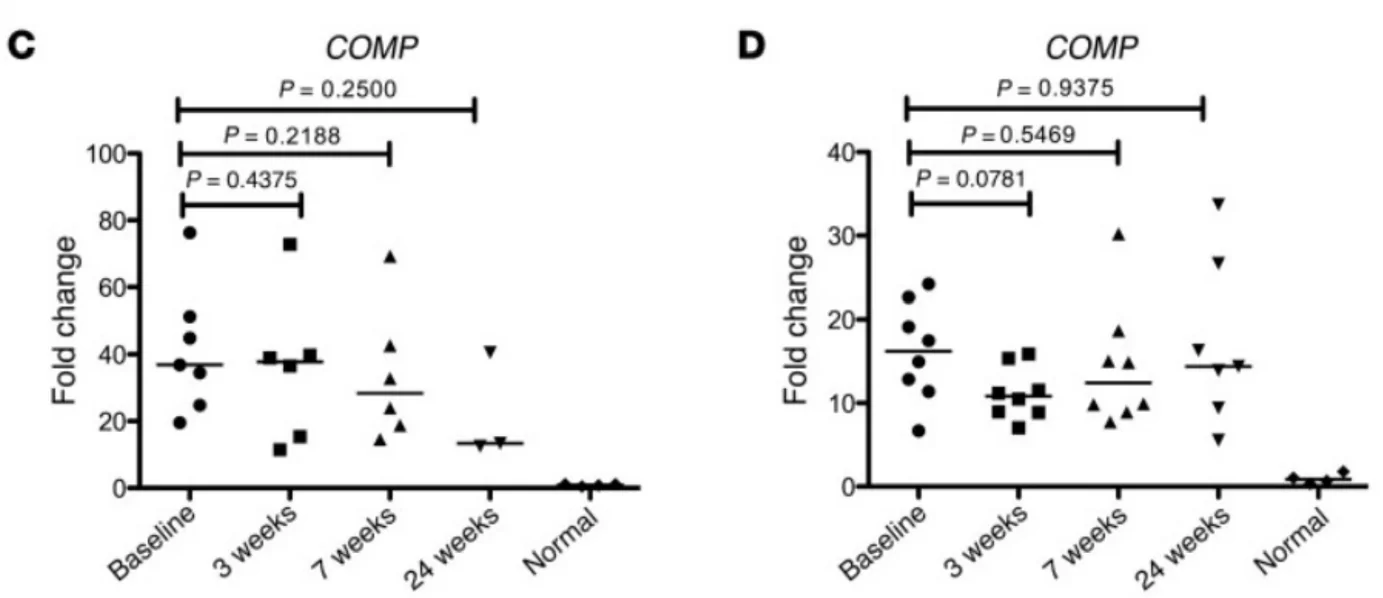
*In vivo* data results taken form Rice et al. (2015)

Assuming that $1\,cm^3$ of tissue has average mass of 1g, we get $c_S = 1 \cdot 10^{-6} g/cm^3$ in Treatment 1, and $c_S = 5 \cdot 10^{-6} g/cm^3$ in Treatment 2. We assume that the initial conditions of the patients are the same as the values at t=168d in Fig. 2, and S(0)=0. Figure 5 taken from Rice et al. (2015) Fig. 3C and D shows the total change of COMP in Treatments 1 and 2.

**Fig. 6:**
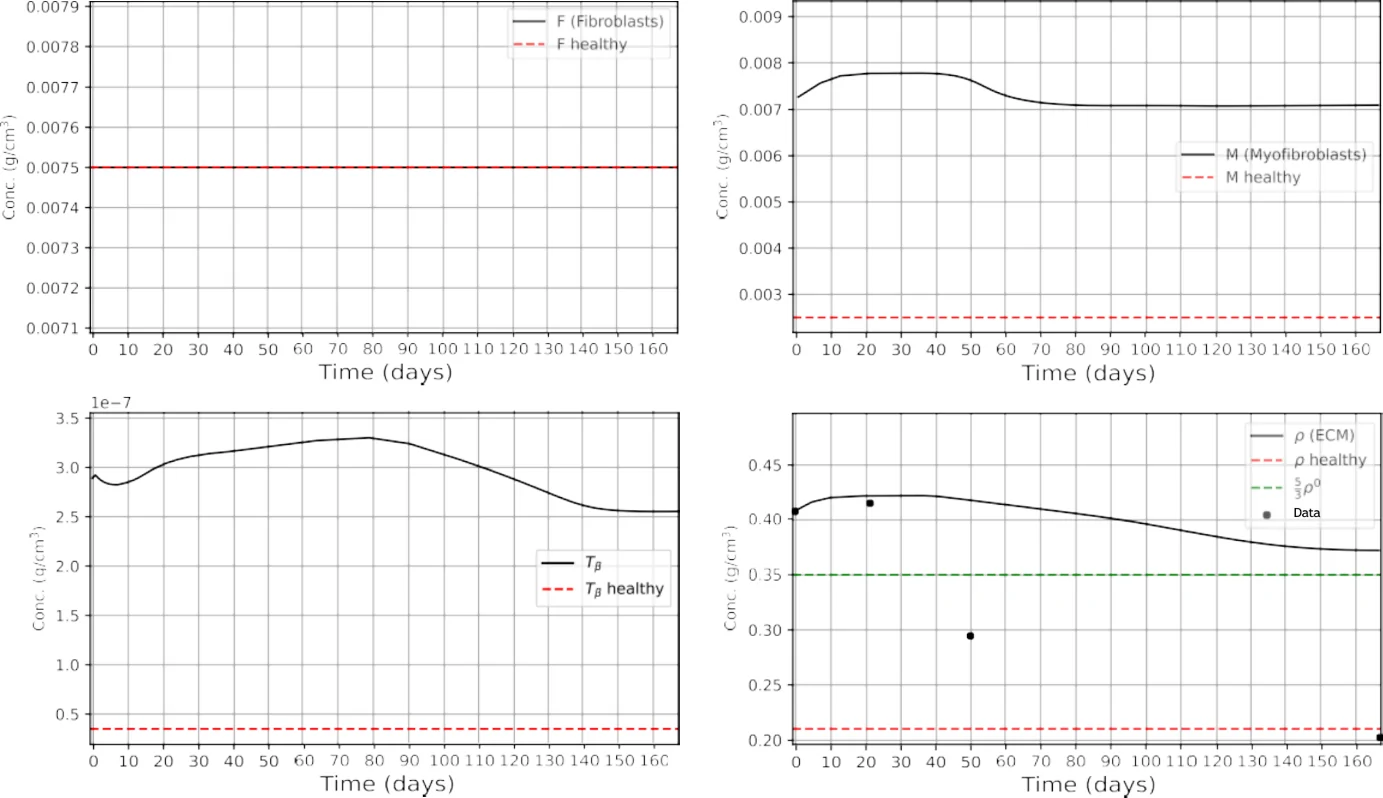
Average densities/concentrations, in $g/cm^3$, of all the model variables for the case of SSc **Treatment 1** with drug S (Color figure online)

**Fig. 7:**
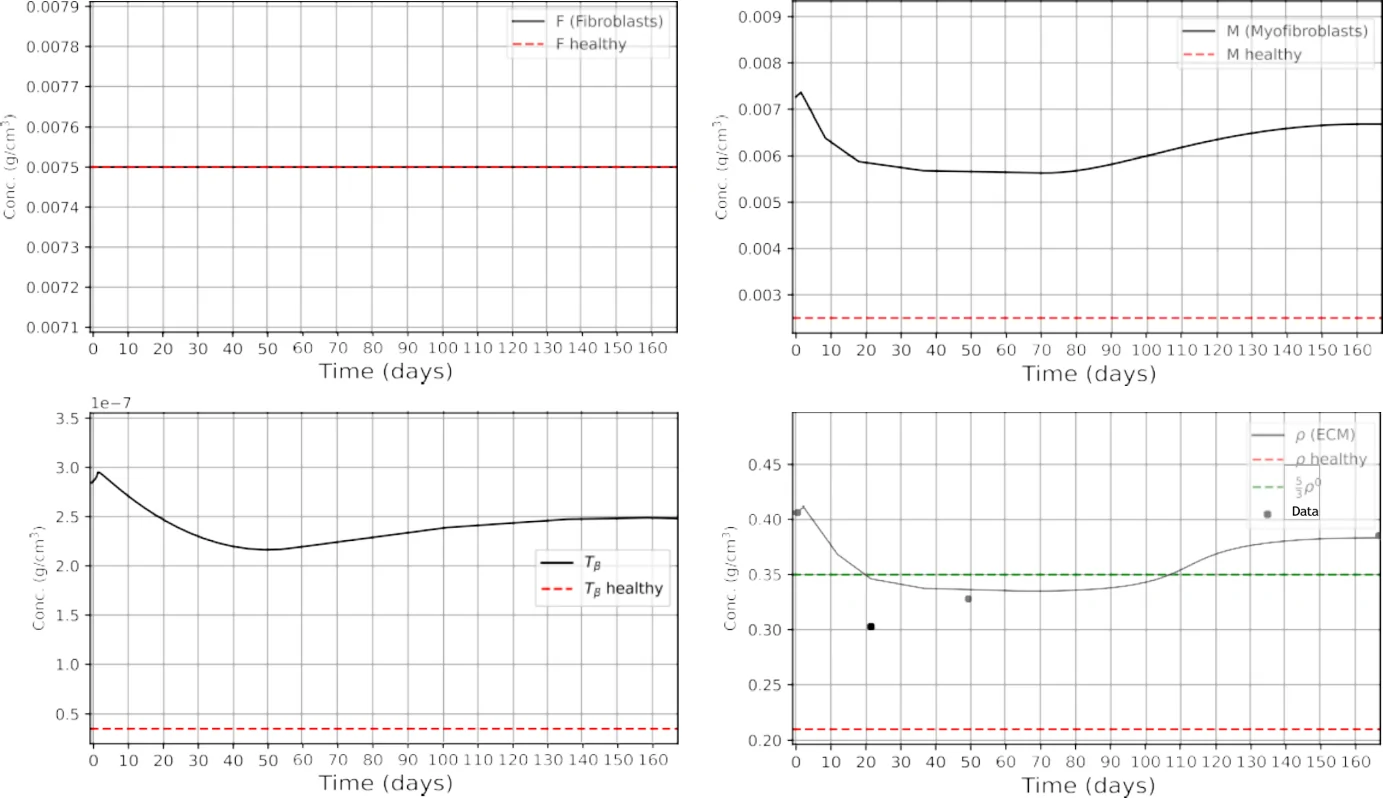
Average densities/concentrations, in $g/cm^3$, of all the model variables for the case of SSc **Treatment 2** with drug S (Color figure online)

In Fig. 6, which simulates Treatment 1, we see that ρ(t) increases initially and then decreases monotonically, in qualitative agreement with COMP gene expression in Rice et al. (2015) Fig. 3C. In Fig. 7, which simulates Treatment 2, we see that ρ(t) is first decreasing and then, after 40 days, it starts to increase monotonically. This behavior is in qualitative agreement with COMP expression in Rice et al. (2015) Fig. 3D. Moreover, comparing the simulation of ρ(t) in Fig. 7 with the 4 data points in Fig. 3D of Rice et al. (2015), we find that the measure of fitness is $R^2 = 0.65$. In the case of Fig. 6, the data point at 24 weeks in Fig. 3C of Rice et al. (2015) is not statistically significant (only 3 patients), and measure of fitness of ρ(t) with the remaining three data points is $R^2 = 0.61$.

**Fig. 8:**
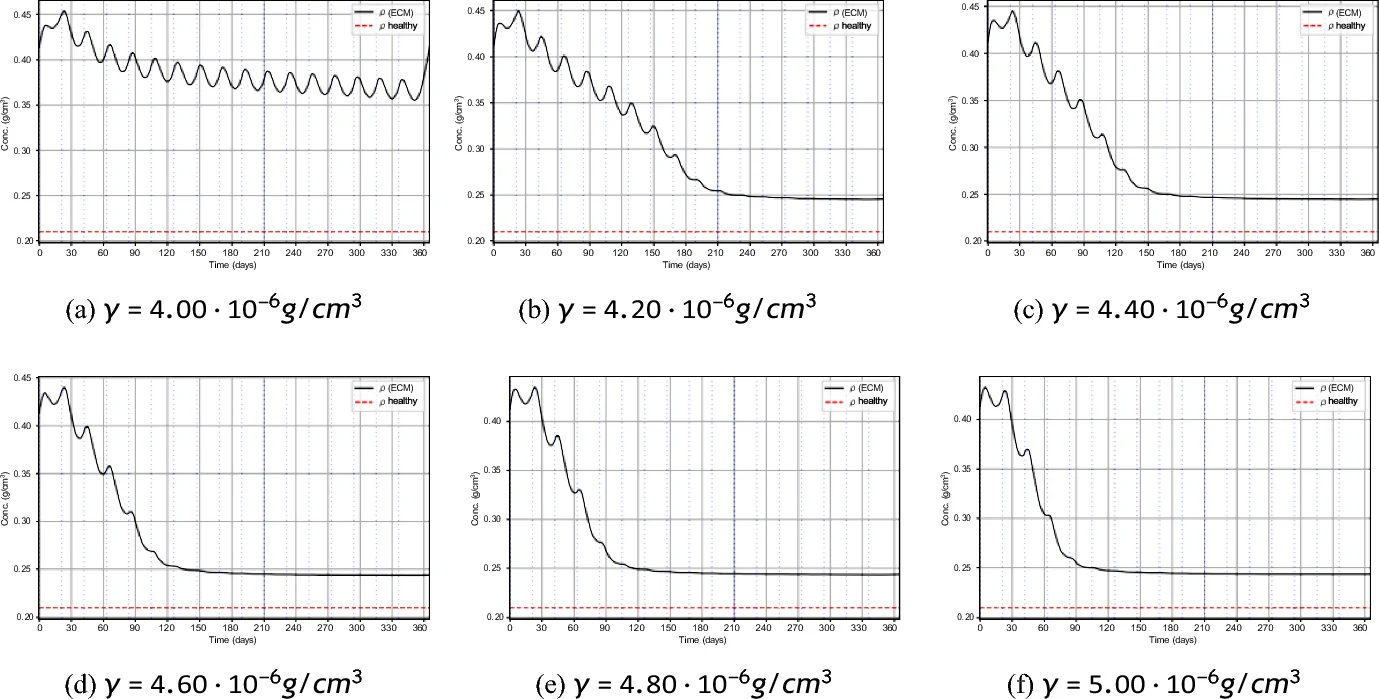
ρ(t) under fractionated fresolimumab dosing with injections every 21 days. Each panel shows a different total dose γ (distributed equally across injections). Periodic dosing induces oscillations; $\gamma =4.0\times 10^{-6}\,\mathrm {g/cm^3}$ yields limited reduction, while larger γ leads to a lower, stabilized plateau after a few months with $\rho (365)=0.25\,\mathrm {g/cm^3}$. Note that $10^{-6}g/cm^3=mg/kg$ (Color figure online)

**Fig. 9:**
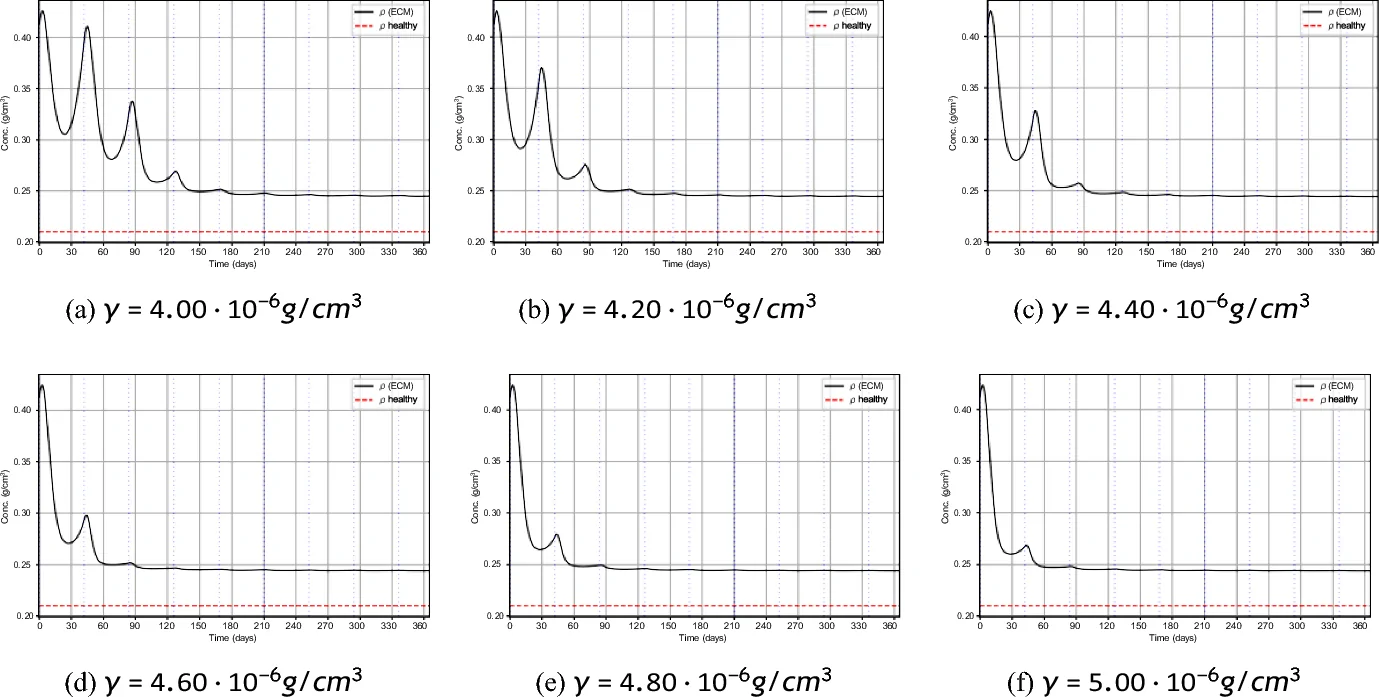
ρ(t) under fractionated fresolimumab dosing with injections every 42 days for total doses γ. Curves show a brief transient with larger oscillations, then converge to small oscillations around a maintained plateau near $\rho (365)=0.25\,\mathrm {g/cm^3}$, with slightly improved stabilization as γ increases. Note that $10^{-6}g/cm^3=mg/kg$ (Color figure online)

**Fig. 10:**
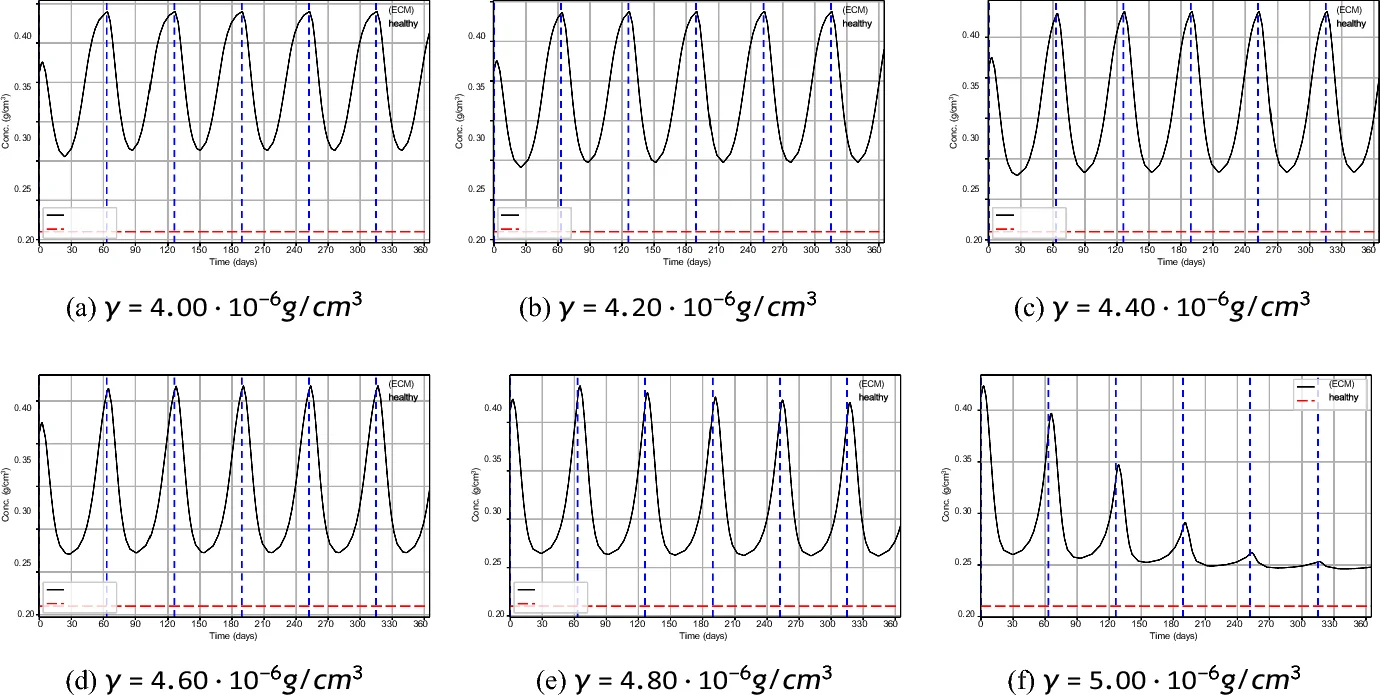
ρ(t) under fractionated fresolimumab dosing with injections every 63 days for total doses γ. Longer spacing produces sustained large oscillations for $\gamma \le 4.8\times 10^{-6}\,\mathrm {g/cm^3}$; at $\gamma =5.0\times 10^{-6}\,\mathrm {g/cm^3}$ oscillations damp and ρ(t) stays closer to $0.25\,\mathrm {g/cm^3}$ by 1 year. Note that $10^{-6}g/cm^3=mg/kg$ (Color figure online)

In Rice et al. (2015), S was administered either twice, in days 1 and 21, at dose 1mg/kg, or just once, in day 1, at a dose 5mg/kg. We can use our model to explore other treatment strategies with S. Motivated by the clinical study in Rice et al. (2015), we consider treatments where instead of administering $5\,mg/kg = 5\cdot 10^{-6}g/cm^3$ in day 1, we administer a drug γ, $\gamma \le 5 \cdot 10^{-6} g/cm^3$, in equal fractions such that adjacent injections are either 21 days apart, or multiple of 21 days apart, in each a half-year. Note, for example, that if a drug γ is administered in fractions that are 21 days apart, then each fraction is γ/8.

Figures  8, 9, and 10 show profiles of ρ(t) with γ increasing from $4 \cdot 10^{-6}$ to $5 \cdot 10^{-6} g/cm^3$m and the spacing between adjacent injections are 21, 42, and 63 days, respectively. In Fig. 8, treatment with $\gamma = 4 \cdot 10^{-6} g/cm^3$ is not effective: ρ(t) keep oscillating and the reduction in ρ(t) is small. In all other cases ρ(t) is oscillatingly decreasing for some time, and then stabilizes at approximately $\rho (365) = 0.25g/cm^3$, which is larger than the health state $\rho ^0 = 0.21 g/cm^3$; as γ increases, the stability of ρ(t) is arrived little earlier and ρ(365) is a little smaller.

Figure  9 shows high oscillations for a short time, followed by very small oscillations around a plateau near $\rho (365) = 0.25g/cm^3$, as in Fig. 8.

In Fig. 10, ρ(t) is contentiously widely oscillating for $\gamma \le 4.8 \cdot 10^{-6} g/cm^3$; when $\gamma = 5 \cdot 10^{-6}$ the oscillations are damp and stays closer to $0.25g/cm^3$.

In choosing a treatment with the best benefits, we must take into account that direct blockade of TGF-β may lead to uncontrolled inflammation (Vistnes 2024).

Since several parameters in Table 2 are estimated or fitted rather than measured directly, we performed a local sensitivity analysis to assess the robustness of the model predictions. For each parameter $p_i$, we computed the normalized local sensitivity index:

11 $$\begin{aligned} S_{p_i}^{Y} = \frac{p_i^0}{Y(p^0)} \frac{ Y(p_i^0(1+\eta ))-Y(p_i^0(1-\eta )) }{ 2\eta p_i^0 } = \frac{ Y(p_i^0(1+\eta ))-Y(p_i^0(1-\eta )) }{ 2\eta Y(p^0) },\nonumber \\ \end{aligned}$$

where $p_i^0$ is the baseline value of the parameter, Y is the model output of interest, and η=0.1. We also repeated the calculation with η=0.05 to verify that the ranking of the most influential parameters was not an artifact of the perturbation size.

Table 3 showed that the treatment predictions are most sensitive to parameters that directly regulate ECM accumulation and the myofibroblast–TGF-β feedback loop. In particular, the largest sensitivity indices were obtained for $\lambda _{\rho M}$, $d_\rho$, $\lambda ^*_{T_\beta M}$, $d_{T_\beta S}$, $d_{S T_\beta }$, and ϵ. This is expected, since these parameters directly determine the rate of ECM deposition, ECM turnover, TGF-β-driven myofibroblast activity, and the response to fresolimumab. Parameters associated with cell diffusion and baseline fibroblast dynamics had smaller sensitivity indices for the averaged ECM outcomes.

**Table 3:** Local sensitivity indices for the main treatment outputs

Parameter	$S_{p_i}^{\rho (365)}$	$S_{p_i}^{\min _t \rho (t)}$	$S_{p_i}^{t_{\textrm{plateau}}}$
$\lambda _{\rho M}$	0.62	0.48	0.18
$d_\rho$	-0.71	-0.55	-0.21
$\lambda ^*_{T_\beta M}$	0.44	0.36	0.16
$d_{T_\beta S}$	-0.38	-0.46	-0.29
$d_{S T_\beta }$	0.24	0.31	0.22
ϵ	-0.52	-0.68	-0.35
$\lambda _{\rho F}$	0.14	0.11	0.05
$\lambda _{M T_\beta }$	0.19	0.15	0.07
$\lambda _{F \rightarrow M}$	0.12	0.09	0.04
$\lambda _F$	0.06	0.04	0.02
$d_F$	-0.05	-0.03	-0.02
$d_M$	-0.21	-0.18	-0.08
$d_{T_\beta }$	-0.17	-0.14	-0.06

## Conclusion

Systemic sclerosis (SSc) is an autoimmune fibrotic skin disease marked by excessive extracellular matrix (ECM) deposited by myofibroblasts. SSc has no cure, and can progress from the skin to the lung and other internal organs, where it may affect the survival rate of patients. Clinical studies aim to reduce ECM density (ρ) of the fibrotic tissue; this may alleviate pain and other negative effects of the disease, and reduce the risk of a severe clinical course of the disease. Since SSc is associated with an abnormally high densities of myofibroblasts, clinical studies focus on decreasing the population of myofibroblasts. Two of the drugs used in these studies are imatinib (N) and fresolimumab (S). Imatinib induces apoptosis in myofibroblasts. Fresolimumab is a TGF-β blocker, which inhibits the induction and proliferation of myofibroblasts.

In this paper, we developed a mathematical model of SSc and used it to assess and analyze the efficacy of treatments with N and S in terms of reduction of ρ. We summarize the main results of the paper as follows:

- One year clinical study in Gordon et al. (2014) demonstrated that daily treatment with N of patients at an average dose of 240 mg reduces fibrosis by 22.4%. Our model simulations are in agreement with this result (Fig. 3), by showing a decrease from initial $\rho =0.428g/cm^3$ to terminal $\rho = \rho (365) = 0.373 g/cm^3$. Interestingly, Fig. 3 shows that ρ(t) decreases to its terminal value of $0.373g/cm^3$ within just two months and remains stable thereafter.
- In Gordon et al. (2014), some patients received 400 mg daily, while others required adjustment to as low as 100 mg. In Fig. 4, we simulated the case where treatment is given at 400 mg ($\rho (365) = 0.308g/cm^3$) or at other doses between 240 mg and 400 mg.
- In 6 month clinical study (Rice et al. 2015), S was administered either at 1 mg/kg in days 1 and 21, or just in day 1 at 5 mg/kg. Our model simulations of these two treatments show the measurement of fitness, $R^2 = 0.65$ and $R^2 = 0.61$ with Rice et al. (2015), respectively.
- We used the model to consider one year treatment in fractions, where the drug S is given every 21 days so that the total dose, γ, does not exceed 5 mg/kg in the first 6 months and in the second 6 months. We found (Fig. 8) that, when $4.2 \le \gamma \le 5$ mg/kg, ρ(t) is oscialltingly decreasing for several months and then stabilizes around $\rho (365) = 0.25g/cm^3$; As γ increases the stabilization occurs a little earlier and ρ(365) is very little decreased. When $\gamma = 4\,mg/kg$, ρ(t) does not stabilize and its decrease is small. Similar results are derived when the drug fractions are 42 days apart (Fig. 9), but, when the fractions are 63 days apart, ρ(t) undergoes continuously high oscillations (Fig. 10).

The model has several limitations: (i) TGF-β is secreted by myofibroblasts (Porte et al. 2021), and it is a key growth factor for myofibroblasts formation (van Caam et al. 2018). Since the etiology of SSc is unknown, while the disease is associated with abnormally large populations of myofibroblasts, we made the assumption that the early event in SSc is an abnormally large amount of TGF-β secretion by myofibroblasts. (ii) Since SSc has no cure, the model distinguishes between the healthy ECM baseline, $\rho ^0 = 0.21g/cm^3$, and the pathological excess ECM, $\rho -\rho ^0$. In Eq. (8), the term $-\epsilon /\sqrt{\rho -\rho ^0}$ is introduced as a phenomenological resistance/homeostatic term acting on the excess ECM in the admissible domain $\rho >\rho ^0$. This term is not intended to represent a specific molecular pathway; rather, it provides a compact way to represent the assumption that treatment reduces excess fibrotic ECM while preserving the healthy ECM scaffold. Similar singular or non-Lipschitz terms are used in mathematical models of finite-time extinction and constrained biological state variables (Iagar 2022; Hoang 2025; Colli et al. 2021; Scarpa and Signori 2021). (iii) ECM degradation is represented phenomenologically by the effective term $d_\rho \rho$. This term aggregates multiple biological processes, including MMP-mediated collagen degradation and ECM remodeling. Since fibrosis may involve both excessive ECM deposition and suppressed ECM degradation (Jinnin 2022; Zhao et al. 2022; Mayorca-Guiliani et al. 2025), a future extension of the model could introduce explicit MMP/TIMP dynamics or disease-dependent ECM degradation rates. Such an extension would require independent measurements of MMP activity, TIMP activity, or collagen degradation biomarkers in order to avoid non-identifiability of the degradation parameters.

Our simulations in Figs. 4 and 9 show that fractional treatments with S yield better reduction of ρ(t) than treatments with N, and should be preferable. However, translating the results of the paper into clinical benefits is challenging due to potentially adverse events. The results of the paper could be useful in the design of future clinical trials aimed at decreasing the exessive ECM in SSc patients.
